# Comparison of the Biological Response of a Head and Neck Carcinoma and a Glioblastoma Cell Line Under Neutron Irradiation with BPA Administration

**DOI:** 10.3390/biology14091252

**Published:** 2025-09-12

**Authors:** Patricia Álvarez-Rodríguez, Cristina Méndez-Malagón, Maribel Porras-Quesada, María Pedrosa-Rivera, Ulli Köster, Ignacio Porras, Javier Praena, Rocío Estrada, Leonor Pérez-Fuentes, Juan Luis Osorio-Ceballos, Carmen Ruiz-Ruiz, Lucie Sancey, María José Ruiz-Magaña

**Affiliations:** 1 Centro de Investigación Biomédica (CIBM Granada), Universidad de Granada, Av. del Conocimiento 19, 18016 Granada, Spain; patriar@ugr.es (P.Á.-R.); cmendez7@ugr.es (C.M.-M.); mcarmenr@ugr.es (C.R.-R.); mjruizm@ugr.es (M.J.R.-M.); 2 Institute for Avanced Biosciences (IAB), Inserm U1209, CNRS UMR5309, Université Grenoble Alpes (UGA), Site Santé, Allée des Alpes, 38000 Grenoble, France; lucie.sancey@univ-grenoble-alpes.fr; 3 Institut Laue-Langevin (ILL), 71 Avenue des Martyrs, 38042 Grenoble, France; 4 Departamento de Bioquímica y Biología Molecular 3 e Inmunología, Facultad de Medicina, Universidad de Granada, Av. Doctor Jesús Candel Fábregas, 11, 18016 Granada, Spain; 5 Labsinlove SL, Avenida del Conocimiento nº 41, 18016 Granada, Spain; 6 Departamento de Física Atómica, Molecular y Nuclear, Facultad de Ciencias, Universidad de Granada, Avda. Fuentenueva s/n, 18071 Granada, Spain; 7 Servicio de Radiofísica y Protección Radiológica, Hospital Universitario Virgen de las Nieves, Av. de las Fuerzas Armadas, 2, 18014 Granada, Spain; 8 Departamento de Biología Celular, Facultad de Ciencias, Universidad de Granada, Avda. Fuentenueva s/n, 18071 Granada, Spain

**Keywords:** BNCT, compound biological effectiveness, boronophenylalanine

## Abstract

Background and objective: In Boron Neutron Capture Therapy, treatment planning is based on a weighted dose in which dose components are weighted with different biological effectiveness (RBE) factors. The most important one for the tumor dose is the compound dependent biological effectiveness (CBE) factor which weighs the boron dose. This was established for brain tumors from radiobiological experiments and has been extrapolated to the more recent application of BNCT to head and neck cancers. The purpose of this work is to study the validity of this assumption. Methods: Two cell lines, CAL-33 (head and neck squamous cell carcinoma) and A172 (Glioblastoma Multiforme), were irradiated with a very pure thermal-equivalent neutron beam after BPA incubation, and the surviving fraction of cells after irradiation was determined by clonogenicity assays. Results: The Compound Biological Effectiveness of both cell lines is similar, although for head and neck carcinoma, 10% higher values are systematically found. In addition to this, radiobiological coefficients for the different dose components (photon, neutron and boron) of BNCT in both cell lines are provided.

## Simple Summary

Boron Neutron Capture Therapy is a promising form of radiotherapy for diffuse tumors, because of its selectivity at the cellular level. This is due to the reaction of thermal neutrons with ^10^B atoms which are selectively delivered to the tumor cells, by means of an appropriate compound. Boronophenylalanine (BPA) is the compound used in the most recent clinical trials. For accurate treatment planning, it is required to include the biological response of the boron reaction by means of a weighting factor in the dose. This weighting factor had been determined for the treatment of brain tumors by different radiobiology experiments and was extrapolated to head and neck cancer treatments because of a lack of specific radiobiology data for the latter. The experimental confirmation or refutation of this hypothesis is still pending. We performed a dedicated experiment of a comparison of the biological response between two cell lines, one of head and neck cancer and another one of a brain tumor, under BPA administration and neutron irradiation to check the validity of the assumption. The results show a similar biological response, with a slightly higher effect of the boron reaction on the head and neck cancer cells for the same dose delivered.

## 1. Introduction

For about 30 years, BNCT was applied only to brain tumors, especially glioblastoma multiforme (GBM) [1]. The first promising clinical results from Hatanaka were obtained with the use of BSH as a boron carrier [2,3]. However, since the 1990s, boronophenylalanine (BPA) [4] has increasingly replaced BSH [5,6] because of its higher tumor selectivity, exploiting the overexpression of amino acid transporters (mainly LAT-1) in tumor cells [7,8,9]. Radiobiological data of the combined boron-neutron effect on tumor cells for brain tumors were obtained by Coderre et al. [10,11] and were used for the treatment of GBM [12,13,14,15,16,17]. In the beginning of the present century, BNCT with BPA was applied to treat head and neck cancers, showing better outcomes than those obtained in brain tumor applications [18,19,20]. Based on the recent results of BNCT with BPA and the new accelerator-based neutron sources developed, the Japanese health system approved BNCT as a therapy for recurrent head and neck cancers in 2020 [21,22], and clinical trials for the same cancers have recently been initiated in Finland [23]. Treatment planning for all these clinical procedures is based on the old data of the RBE factors obtained for brain tumors, including the CBE factor used for the boron dose which is the main component of the tumor dose [24]. It is of particular interest to check whether this extrapolation is adequate or not for head and neck cancers. To date, the only study addressing this topic is the work of Bernardini et al., in which photon isoeffective dose curves for head and neck carcinoma were obtained, and its CBE value was calculated [25]. Although this study offers pioneer and valuable information, our work aims to provide a more realistic comparison by evaluating the effects of BNCT on both head and neck carcinoma and GBM cell lines under identical experimental conditions, allowing for a direct comparison of CBE values between these different tumor types. To do so, the PF1b neutron line [26] at the Institute Laue-Langevin (ILL, Grenoble, France) is perfect for this test as it produces a thermal-equivalent neutron beam with negligible contamination of either fast neutrons or gammas [27,28]. With an appropriate setup, it is possible to irradiate cells treated with boron compounds and analyze their survival in a biological laboratory placed in the same experimental hall. Using this neutron beamline and thanks to a collaboration between the University of Granada, the University of Grenoble-Alps and ILL, a set of measurements was performed to assess the response of BPA-loaded cells to neutron irradiation. The results of these measurements are presented in this paper.

## 2. Materials and Methods

### 2.1. Cell Culture

Irradiations were performed on two cell lines of human origin, both obtained from collections. CAL-33 cells derive from head and neck squamous cell carcinoma and A172 cells from glioblastoma multiforme. CAL-33 cells were kindly provided by the Institute for Advanced Biosciences, Grenoble, and A172 cells were provided by the Department of Human Anatomy and Embryology from the University of Granada.

The culture medium used was DMEM (Gibco, Carlsbad, CA, USA) for both cell lines. This medium was supplemented with 10% heat-inactivated fetal bovine serum (FBS; Gibco) and 1% penicillin and streptomycin (Gibco). The heat inactivation of the FBS was performed at 56 °C for 30 min and both cell lines were cultured at 37 °C in a humidified environment containing 5% CO_2_ (Galaxy^®^ 48R/48S CO_2_ Incubator, Eppendorf, Hamburg, Germany). The experiments were performed with cells between passages 9 and 14.

### 2.2. Boron Compound Uptake

The compound used to deliver ^10^B into the cells was ^10^B-enriched boronophenylalanine (BPA) (Interpharma, Praha, Czech Republic). BPA was dissolved in fructose (Sigma-Aldrich, St. Louis, MO, USA) and added to the culture media at a final concentration of 80 ppm. Both A172 and CAL-33 cells were incubated in presence of BPA for 4 h prior to irradiation to ensure its uptake by the cells.

Boron concentration within the cells was measured by ICP-AES (Inductively Coupled Plasma Atomic Emission Spectroscopy, Perkin Elmer OPTIMA 8300, Perkin Elmer, Shelton, CT, USA) at the Centro de Instrumentación Científica of the University of Granada. Treatments were performed when cultures reached 80–90% confluence, in T75 culture flasks (Sigma-Aldrich). After 4 h incubation in presence of BPA, the cells were detached with 1% trypsin-EDTA (Sigma-Aldrich), rinsed with PBS to remove any extracellular boron traces and then dried. The cells were subsequently weighed and digested in a 65% nitric acid solution (Sigma-Aldrich) for 60 min at 100 °C. Prior to the measurements, a calibration curve with known quantities of boron was prepared, from which the final boron uptake of each sample was obtained. The results were expressed in terms of parts per million (ppm), adjusted according to the weight of each sample.

### 2.3. Nitrogen Content Measurement

The nitrogen content of the cells, which is important to estimate the neutron dose, was measured for both cell lines by elemental analysis at the Centro de Instrumentación Científica of the University of Granada. Cells were detached and thoroughly washed several times with PBS to remove any residual culture medium or extracellular components. Subsequently, the cell pellets were briefly dried using a thermoblock prior to elemental analysis. Afterwards, the percentages of carbon, nitrogen, hydrogen and sulfur were determined by dynamic combustion in a CHNS Elemental analyzer (Thermo Scientific Flash 2000, Thermo Fisher Scientific, Alcobendas, Spain).

### 2.4. Neutron Irradiations

Neutron irradiations were performed at the PF1b cold neutron beamline of the Institut Laue-Langevin (ILL, Grenoble, France), with fluxes on the order of 10^9^ n∙s^−1^∙cm^−2^, which vary between reactor cycles. The energy of these cold neutrons is lower than that of thermal neutrons, but the capture effects and neutron doses are equivalent as previously detailed by Pedrosa-Rivera et al. [27]. The PF1b line provides a pure thermal-equivalent neutron beam, since the fast neutron and gamma contributions are negligible due to the bent neutron guide, which makes it ideal for studying the isolated effects of thermal-equivalent neutrons.

**Figure 1:**
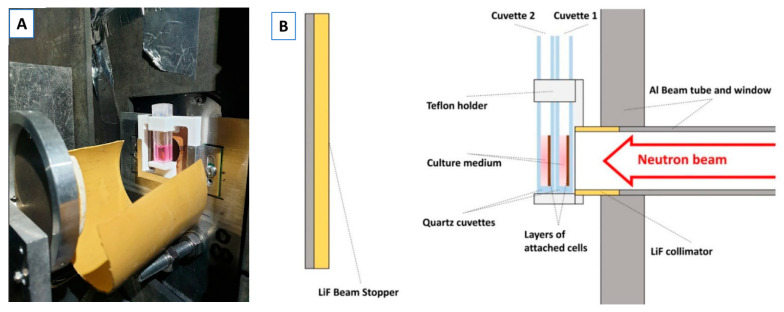
Experimental setup for cell irradiation at ILL. Picture (**A**) and scheme (**B**) of the experimental setup used in the PF1b instrument to perform neutron irradiation at ILL. The scheme shows the position of the cuvettes and the neutron beam orientation. Two cuvettes with cells were positioned and irradiated simultaneously and due to neutron beam attenuation, the cuvette 1 placed closer to the beam received a higher dose than the cuvette 2 [29].

The experimental setup, described in a previous work [29], allows two samples to be irradiated simultaneously (Figure 1). For irradiation, cells were placed in culture media inside quartz cuvettes with a 2 mm-thick layer of culture medium, where they naturally attach as a monolayer after 24 h. These cuvettes were positioned perpendicular to the neutron beam for the irradiation, so that due to the neutron beam attenuation, the one closer to the beam receives more dose than the one located farther away.

Prior to irradiation, the thermal equivalent flux was measured by activation of Au foils. Then, the experiments were carried out in at least triplicates for each dose, with irradiation times ranging from 15 to 75 min in absence of BPA and from 1 to 7 min in presence of BPA.

To avoid boron loss in the cells during the irradiation and for a realistic determination of the boron dose received, the medium with the BPA was maintained. As the boron concentration was superior in the medium than in the cells, the excess of boron dose due to alpha particle disequilibrium (fraction of the alpha particle dose deposited inside the cells from outside emissions that is greater than the dose leaking to the exterior from captures occurring within the cells) was calculated by Monte Carlo simulations and included in the calculations by means of a correction factor.

### 2.5. Photon Irradiations at the Medical LINAC

Photon irradiations were carried out using an Elekta Versa HD™ 6 MV medical linear accelerator (LINAC) at the University Hospital Virgen de las Nieves (HUVN) in Granada. We chose this beam as the reference photon radiation because it is a standard real beam used in radiation therapy, and the aim of the photon isoeffective dose is to compare the biological effects with this conventional therapy, more representative of current clinical treatments than other photon sources used in radiobiological experiments such as 60Co sources or X-ray irradiators.

**Figure 2:**
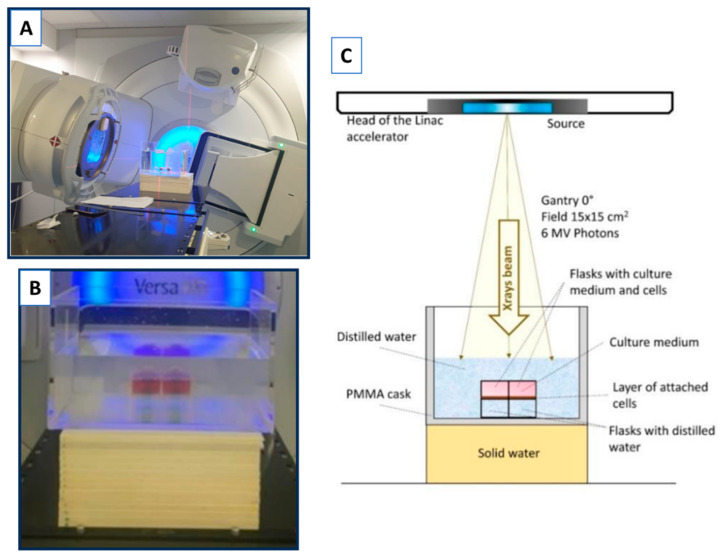
Experimental setup for photon irradiation using the LINAC. (**A**) Picture of the LINAC used for photon irradiation at the University Hospital Virgen de las Nieves (HUVN) in Granada. (**B**) Zoomed view of the experimental setup showing the arrangement of T25 flasks containing cells. (**C**) Schematic representation of the setup illustrating flasks positioning and beam direction. Two flasks containing cells were irradiated at the same time, receiving the same dose [28,29].

Cells were seeded in T25 cell culture flasks (Sigma-Aldrich). To ensure the electronic equilibrium, the flasks were filled with culture medium, immersed in distilled water, and placed on 10 cm of solid water (Figure 2) [28,29]. Two flasks were simultaneously irradiated, each receiving the same dose. The tested doses ranged from 0.5 to 8 Gy, delivered at a dose rate of 1 Gy/min.

Photon doses were calculated using a computed tomography scan of the setup, which was processed using the Pinnacle treatment planning system v.16.2.1 (Philips, Amsterdam, The Netherlands). This enabled the selection of the optimal field size to ensure cells were homogeneously irradiated.

### 2.6. Clonogenic Assay

After irradiation, cells were detached using 1% trypsin-EDTA and used to carry out clonogenic assays. For these studies, cells were seeded in triplicate in six-well plates. The number of cells seeded (between 400 and 6000 per well) depends on the dose absorbed by those cells and their growth characteristics. Then, cells were incubated for 8–10 days to allow for the formation of colonies visible to the naked eye. Every four days, medium was exchanged with fresh medium. Once the colonies were formed, they were fixed with 90% ethanol and stained with crystal violet (Sigma-Aldrich), and those with more than 50 cells were counted.

Afterwards, the plating efficiency (PE) of each sample was calculated. The PE reflects the ability of the cells to proliferate and form colonies. It is expressed as the ratio between the number of colonies counted and the number of cells seeded in each sample:

$${PE}_{i}=\frac{number\;of\;colonies\;counted\;(i)}{number\;of\;cells\;seeded\;(i)}\;,$$

where (i) refers to the condition studied. The survival of each sample was calculated from the PE values obtained by comparing the PEs of each condition and the control, as indicated by the following equation, so the survival of the control sample is always 1 [30].

$$S_{i}=\frac{{PE}_{i}}{{PE}_{CT}}$$

### 2.7. Determination of CBE

To obtain the Compound Biological Effectiveness (CBE) values for BPA in both cell lines, it is necessary to obtain the survival curves after irradiation. We adopted the formalism of an approximation to the photon isoeffective dose as we previously described [31,32]. The data obtained from the neutron irradiations were fitted by the well-known linear-quadratic formula [33]:

(1) $$S=e^{-\alpha_{n}D_{n}-\alpha_{B}D_{B}-\alpha_{\gamma }D_{\gamma }-\beta_{\gamma }D_{\gamma }^{2}},$$

where the effect of the different dose components is considered. $D_{n}$ stands for the pure neutron dose (excluding the boron reaction and secondary gammas), $D_{B}$ denotes the dose delivered locally by the neutron-boron reaction (alpha particle and recoil lithium) and $D_{\gamma }$ accounts for the dose delivered by secondary photons produced by the neutrons in the medium and cells (the beam itself has negligible gamma contamination). We assumed, as can be confirmed in the Section 3, that the quadratic term of the neutron and boron dose can be neglected since both are due to high-LET (Linear Energy Transfer) radiation. Also, the photon Lea-Catcheside factors were assumed as 1. This is clear for glioblastoma cells, for which the repair times, as reported by Marcaccio et al. [24], are much longer than the irradiation time in our experiments. Another assumption is that synergies between the different dose components have been neglected as in our experiments these are very different in magnitude and therefore difficult to observe its potential effect.

The CBE is obtained by a comparison of the effect of the neutron-boron reaction, given by the factor

(2) $${S_{B}=e}^{-\alpha_{B}D_{B}}\;,$$

with the effect of a reference conventional photon irradiation, which we called

(3) $$S_{0}=e^{-\alpha_{\gamma }D_{0}-\beta_{\gamma }D_{0}^{2}}$$

As there is no data on the specific radiobiological coefficients for the photon component in the neutron irradiations (mainly photons of 2.2 MeV), we assumed here that we can approximate them by the radiobiological coefficient obtained from the irradiations at the medical LINAC, from the fit:

(4) $$S_{\gamma }=e^{-\alpha_{\gamma }D_{\gamma }-\beta_{\gamma }D_{\gamma }^{2}}\;,$$

where $S_{\gamma }$ denotes the survival after irradiation at the LINAC at dose $D_{\gamma }$. The mean energy of the photons from the LINAC depends on the beam size, the reference point and depth, but for the 6 MV one, it has been reported [34] to lie between 1.3 and 1.7, not too far from the 2.2 MeV of the hydrogen capture photons in neutron irradiations. Moreover, the photon RBE has been shown to be very insensitive to the photon energies from 1 to 10 MeV and very close to 1 [35], which justify our assumption.

The different coefficients were obtained as follows. First, the coefficients $\alpha_{\gamma },\beta_{\gamma }$ were calculated by a fit to Equation (4) of the values of the survival data obtained from the photon irradiations at the medical LINAC. Then, using the previously found $\alpha_{\gamma },\beta_{\gamma }$, a fit of the survival data from the neutron irradiations without boron administration was performed via the formula:

(5) $$S_{ILL}=e^{-\alpha_{n}D_{n}-\alpha_{\gamma }D_{\gamma }-\beta_{\gamma }D_{\gamma }^{2}},$$

from which the coefficient $\alpha_{n}$ is obtained. $S_{ILL}$ denotes the survival after irradiation with the ILL beam (with no boron administration), which delivers a pure neutron dose $D_{n}$ and a secondary induced photon dose $D_{\gamma }\;.$

The remaining $\alpha_{B}$ coefficient was determined by fitting the cell survival data under neutron irradiation and boron administration to Equation (1).

Finally, the CBE was calculated by comparing a given dose from the boron-neutron reaction, ${D_{B}}^{\left(0\right)}$, and the dose from a reference photon irradiation, $D_{0}$, which produces the same biological effect:

(6) $$e^{-\alpha_{B}{D_{B}}^{\left(0\right)}}=e^{-\alpha_{\gamma }D_{0}-\beta_{\gamma }D_{0}^{2}}\;,$$

which leads to:

(7) $$D_{0}=\frac{\alpha_{\gamma }}{2\beta_{\gamma }\;}\left(\sqrt{1+4\frac{\alpha_{B}\beta_{\gamma }}{{\alpha_{\gamma }}^{2}}{D_{B}}^{\left(0\right)}}-1\right)$$

The CBE factor is then given by:

(8) $$CBE=\frac{D_{0}}{{D_{B}}^{\left(0\right)}}$$

All fittings were performed using the Levenberg–Marquardt algorithm as described in [36], considering the errors in the data.

## 3. Results

### 3.1. Nitrogen Content and Boron Uptake Coefficients

The data on nitrogen content and on the boron concentration achieved in cells following incubation with BPA (at 80 ppm for 4 h) are illustrated in Table 1. These data are required for a proper evaluation of the neutron dose (thermal, also called nitrogen dose) and for the boron dose, respectively. GBM cells have more nitrogen, and the boron uptake is slightly smaller than that for head and neck tumor cells.

### 3.2. Photon Radiobiology Coefficients

**Figure 3:**
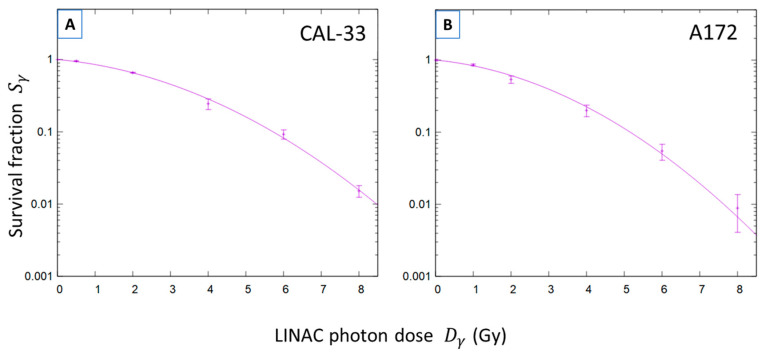
Survival curves of CAL-33 and A172 cells after photon irradiation at HUVN. (**A**) Survival curve of CAL-33 cells after photon irradiation at the medical LINAC. (**B**) Survival curve of A172 cells after photon irradiation at the medical LINAC. Data points represent experimentally measured survival ($S_{\gamma }$), and the purple line corresponds to the fitted survival curve.

Survival of CAL-33 and A172 cells under photon irradiation in the medical LINAC is illustrated in Figure 3, where the best fits by Equation (4) are also displayed with lines. The radiobiology coefficients $\alpha_{\gamma },\;\beta_{\gamma }$ obtained are shown in Table 2. For the CAL-33 cell line, our values, with more statistics than in our previous measurement reported in [26], are in close agreement with the results of Bauer et al. [37]. For the A172 cells, we found a discrepancy when compared with another glioblastoma cell line (U87), and for the alpha coefficient, a value of 0.21 ± 0.08 was obtained [24]. Nevertheless, there is some overlap within the uncertainties of our result. This highlights the importance of reducing uncertainties in radiobiological measurements.

### 3.3. Neutron Radiobiology Coefficients

**Figure 4:**
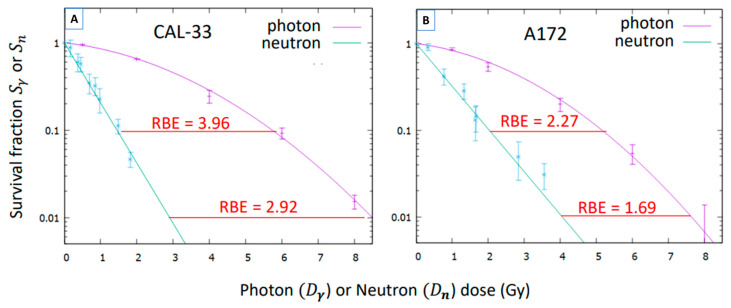
Survival fraction of CAL-33 and A172 cells exposed to the pure neutron irradiation $S_{n}$ as a function of the neutron dose $D_{n}$, compared to their survival under photon exposure. (**A**) Survival curve of CAL-33 cells after neutron irradiation at ILL compared to the survival after photon irradiation. (**B**) Survival curve of A172 cells after neutron irradiation at ILL compared to the survival after photon irradiation. Data points represent experimentally measured survivals ($S_{n}$ and $S_{\gamma }$), and the lines corresponds to the fitted survival curves (green: neutrons, purple: photons). The neutron RBE at two different survival fractions is highlighted in red. The estimated uncertainties of the RBE are 10% for the CAL-33 cells and 30% for the A172 ones.

The survival fractions under neutron irradiation, $S_{ILL}$, were measured for both cell lines. Cell killing results from the combined effect of a pure neutron dose and an induced gamma dose arising from radiative captures in the materials. To separate both effects (assuming $S_{ILL}{=S}_{n}{\;S}_{\gamma }$), we plotted (using the previous $\alpha_{\gamma },\beta_{\gamma }$ values of Table 2) the function.

(9) $$S_{n}{=S}_{ILL}\;e^{\alpha_{\gamma }D_{\gamma }+\beta_{\gamma }D_{\gamma }^{2}},$$

whose values were fitted to the function $e^{-\alpha_{n}D_{n}}$, according to Equation (5). These fits are illustrated in Figure 4 and compared to the survival under photons. The values of the parameter $\alpha_{n}$ obtained are displayed in Table 3, where the values for the coefficients $W_{n}$ for the formalism of Ref. [31] are also shown.

### 3.4. Compound Biological Effectiveness (CBE)

**Figure 5:**
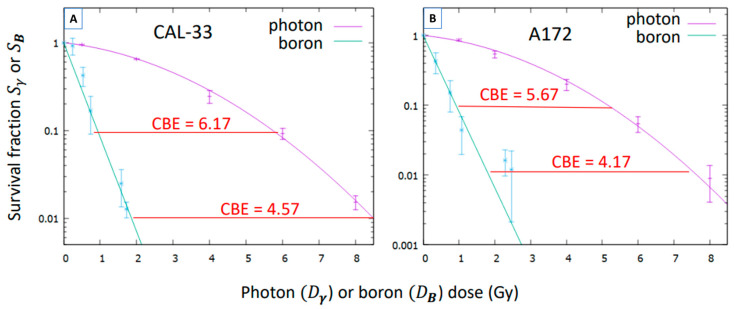
Survival fraction of CAL-33 and A172 cells by the pure boron effect $S_{B}$ as a function of the boron dose $D_{B}$, compared to their survival under photon irradiation. (**A**) Survival curve of CAL-33 cells after neutron irradiation in presence of BPA, compared to their survival under photon irradiation. (**B**) Survival curve of A172 cells after neutron irradiation in presence of BPA, compared to their survival under photon irradiation. Data points represent experimentally measured survival ($S_{B}$ and $S_{\gamma }$), and the lines correspond to the fitted survival curves (green: BPA-neutrons, purple: photons). The values of the CBE at two different survival fractions are highlighted in red. Their estimated uncertainties are 12% for the CAL-33 cells and 33% for the A172 ones.

From the measured cell survival S after BPA incubation and neutron irradiation, we estimate the pure boron survival $S_{B}\;$ by means of:

(10) $$S_{B}=S\;e^{{\alpha_{n}D_{n}+\;\alpha }_{\gamma }D_{\gamma }+\beta_{\gamma }D_{\gamma }^{2}},$$

using the parameters ${\alpha_{n},\;\alpha }_{\gamma },\;\beta_{\gamma }$ reported above and where $D_{n},\;D_{\gamma }$ are the dose components of these irradiations. Then, a fitting of the function $e^{-\alpha_{B}D_{B}}$ to the values of $S_{B}$ was performed (Figure 5), which provides the values in Table 3.

In the calculation of the boron dose $D_{B}$, the boron uptake values reported in Table 1 were used, together with a dose enhancement factor due to the boron captures from the excess of BPA in the medium: 1.7 for A172 cells and 1.5 for CAL-33 cells (obtained from Monte Carlo simulations). The values for the coefficients $W_{B}$ for the formalism of Ref. [31] are also shown in Table 3.

The coefficient $\alpha_{n}$ for the CAL-33 cells previously published in [29] was slightly corrected by the inclusion of new data (1.585 vs. 1.65).

Finally, the CBE, as a function of the boron dose, can be estimated by using Equations (7) and (8). The results are illustrated in Table 4. For illustration, in Figure 5 the CBE values are also shown at two different survival fractions.

## 4. Discussion

To the best of our knowledge, this is the first study in vitro aiming to obtain and compare the CBE factors of head and neck carcinoma and GBM using BPA as boron delivery agent under identical irradiation conditions. Previous studies have reported CBE values for these tumor types [10,25], but they were conducted separately and under different experimental settings. In contrast, our approach ensures a controlled comparison that allows for a more reliable interpretation of the differences in the biological responses of both types of tumors within the same experimental framework.

Interestingly, from the results presented in Table 4, it can be noticed that the CBE values are very similar for both cell lines, which suggests that the extrapolation of the coefficients used in BNCT trials of GBM to head and neck cancers seems reasonably valid. However, we observed a systematic 10% increase in these values for head and neck cancer that may partly explain the higher efficacy of the treatment for these cancers (in addition to the higher BPA uptake). This could be possibly due to a slightly more effective microdistribution inside the cell. Other factors, such as differences between both tumor types and selected cell lines in the extent of DNA damage induction and their repair capacity, may also play an important role. On the other hand, there are some uncertainties difficult to estimate (alpha particle disequilibrium, BPA uptake values, etc.) that may require further experimentation to reduce them and to ascertain whether the differences found are statistically significant.

The results shown in Figure 5 demonstrate that BNCT has greater biological effectiveness compared to conventional photon irradiation. This highlights its enhanced therapeutic potential and supports its promising application in clinical oncology, especially in cases that require high tumor selectivity and effective local control.

The CBE value obtained for head and neck carcinoma (4.57) is consistent with previous findings reported by Bernardini et al. (4.3) [25], despite differences in the cellular model and experimental settings, which reinforces the robustness of the observed effect.

The present study was performed in vitro, which offers valuable information. The next step would be to explore in vivo the CBE factors obtained and to evaluate the correlation between both conditions, taking into account the tumor environment complexity that cannot be reproduced in vitro. These preclinical animal studies and future clinical trials in patients will be essential to verify the results presented here and to allow their translation into daily clinics.

Finally, although the CBE extrapolation in these two cases might be feasible, it cannot be systematically applied to every condition. It should be considered that the CBE is not a fixed value, but it depends on several parameters such as dose, cell type and boron distribution. Therefore, this assumption should be carefully evaluated not only for other types of tissues but also for different cell lines of the same tumor type, for which the CBE factors should be determined. This would contribute to optimizing future BNCT treatment plannings.

## 5. Conclusions

The present study provides valuable information on the CBE factors for head and neck carcinoma and GBM under identical irradiation conditions. The similarity observed between both tumor types suggests that CBE extrapolation from GBM to head and neck carcinoma may be reasonable, although with a systematic increase for the latter that could explain its higher treatment efficacy. The next step is to evaluate these findings in vivo to confirm these results and support their translation into clinical practice.

In addition, the data provided here may be useful for implementation on radiobiological models and models for predicting tumor control, as the contributions from different dose components (photon, thermal neutron and boron) were effectively separated.

## Figures and Tables

**Table 1:** Nitrogen content and boron concentration for the cell lines studied.

Cell Line	%Nitrogen	B Conc (ppm)
CAL-33	2.3 ± 0.1	29 ± 5
A172	3.5 ± 0.5	23 ± 5

**Table 2:** Photon radiobiology coefficients for CAL-33 and A172 cells, obtained from fitting Equation (4) (illustrated in Figure 3).

Cell Line	$\alpha_{\gamma }$ (Gy^−1^)	$\beta_{\gamma }$ (Gy^−2^)	$\alpha_{\gamma }/\beta_{\gamma }$ (Gy)
CAL-33	0.109 ± 0.009	0.051 ± 0.003	2.12 ± 0.30
A172	0.121 ± 0.031	0.063 ± 0.008	1.92 ± 0.74

**Table 3:** Radiobiological coefficients for neutron and boron dose components.

Cell Line	$\alpha_{n}$ (Gy^−1^)	$W_{n}=\alpha_{n}/\alpha_{\gamma }$	$\alpha_{B}$ (Gy^−1^)	$W_{B}=\alpha_{B}/\alpha_{\gamma }$
CAL-33	1.59 ± 0.05	14.5 ± 1.6	2.48 ± 0.11	22.7 ± 2.9
A172	1.02 ± 0.06	8.4 ± 2.6	2.51 ± 0.29	20.8 ± 7.7

**Table 4:** Isoeffective photon doses $D_{0}$ for different boron doses $D_{B}$ and the corresponding values of the CBE, obtained as their ratio, for the two cell lines studied, with an estimated uncertainty of 17% for the CAL-33 and 47% for the A172 cells.

	CAL-33		A172	
$D_{B}(Gy)$	$D_{0}(Gy)$	*CBE*	$D_{0}(Gy)$	*CBE*
1	5.96	5.96	5.43	5.43
2	8.82	4.41	8.02	4.01
3	11.0	3.67	10.0	3.34
4	12.9	3.22	11.7	2.93
5	14.5	2.90	13.2	2.64
6	16.0	2.66	14.5	2.42
7	17.3	2.48	15.8	2.25
8	18.6	2.33	16.9	2.12
9	19.8	2.20	18.0	2.00
10	20.9	2.09	19.0	1.90

## Data Availability

The original contributions presented in this study are included in the article. Further inquiries can be directed to the corresponding authors.
